# A comparison of inducible, ontogenetic, and interspecific sources of variation in the foliar metabolome in tropical trees

**DOI:** 10.7717/peerj.7536

**Published:** 2019-09-20

**Authors:** Brian E. Sedio, Armando Durant Archibold, Juan Camilo Rojas Echeverri, Chloé Debyser, Cristopher A. Boya P, S. Joseph Wright

**Affiliations:** 1 Smithsonian Tropical Research Institute, Balboa, Ancón, Republic of Panama; 2 Center for Biodiversity and Drug Discovery, Instituto de Investigaciones Científicas y Servicios de Alta Tecnología-AIP, Ciudad del Saber, Republic of Panama; 3 Department of Integrative Biology, University of Texas at Austin, Austin, TX, United States of America; 4 Department of Biology, McGill University, Montréal, Québec, Canada; 5 Department of Biotechnology, Acharya Nagarjuna University, Guntur, India

**Keywords:** Intraspecific variation, Mass spectrometry, Coexistence, Plant-herbivore interactions, Metabolomics, Community ecology

## Abstract

Plant interactions with other organisms are mediated by chemistry, yet chemistry varies among conspecific and within individual plants. The foliar metabolome—the suite of small-molecule metabolites found in the leaf—changes during leaf ontogeny and is influenced by the signaling molecule jasmonic acid. Species differences in secondary metabolites are thought to play an important ecological role by limiting the host ranges of herbivores and pathogens, and hence facilitating competitive coexistence among plant species in species-rich plant communities such as tropical forests. Yet it remains unclear how inducible and ontogenetic variation compare with interspecific variation, particularly in tropical trees. Here, we take advantage of novel methods to assemble mass spectra of all compounds in leaf extracts into molecular networks that quantify their chemical structural similarity in order to compare inducible and ontogenetic chemical variation to among-species variation in species-rich tropical tree genera. We ask (i) whether young and mature leaves differ chemically, (ii) whether jasmonic acid-inducible chemical variation differs between young and mature leaves, and (iii) whether interspecific exceeds intraspecific chemical variation for four species from four hyperdiverse tropical tree genera. We observed significant effects of the jasmonic acid treatment for three of eight combinations of species and ontogenetic stage evaluated. Three of the four species also exhibited large metabolomic differences with leaf ontogenetic stage. The profound effect of leaf ontogenetic stage on the foliar metabolome suggests a qualitative turnover in secondary chemistry with leaf ontogeny. We also quantified foliar metabolomes for 45 congeners of the four focal species. Chemical similarity was much greater within than between species for all four genera, even when within-species comparisons included leaves that differed in age and jasmonic acid treatment. Despite ontogenetic and inducible variation within species, chemical differences among congeneric species may be sufficient to partition niche space with respect to chemical defense.

## Introduction

A substantial proportion of the local species richness of many tropical forests is comprised of a small number of exceptionally species-rich woody plant genera (Gentry, 1982). Theory maintains that species must exploit distinct niches defined by resource use or biotic interactions in order to stably coexist, that is, to avoid competitive exclusion of some species from the community (Chesson & Kuang, 2008). High local species richness of congeneric plants poses a challenge to our understanding of diversity maintenance in tropical forests because closely related species are likely to share natural enemies (Novotny et al., 2002; Odegaard, Diserud & Ostbye, 2005; Gilbert & Webb, 2007) and therefore density-dependent recruitment limitation that should result in competitive exclusion of related species and preclude closely related species from coexisting ecologically (Sedio & Ostling, 2013). However, species niche differences defined by secondary metabolites might contribute to the local diversity of species-rich tree genera if congeneric species differ chemically sufficiently to avoid sharing natural enemies (Salazar, Jaramillo & Marquis, 2016; Forrister et al., 2019).

Assessing the potential for secondary metabolites to define niche differences among plant species is complicated by ecological variables that drive chemical differences within and among conspecific individuals. Sedio et al. (2017) found that intraspecific chemical variation due to leaf ontogeny, light environment, and precipitation seasonality was much less than interspecific variation in four species-rich tropical tree genera of tropical moist forest in Panama. However, Sedio et al. (2017) did not consider inducible variation in the metabolome, and other studies of inducible chemical variation in tropical trees have been limited to seasonal, deciduous forests (Boege, 2004, but see Bixenmann et al., 2016).

Plant metabolomes are regulated by signaling pathways governed, in part, by compounds in the oxylipin class of signaling molecules, of which jasmonic acid is a key member. Jasmonic acid and its methyl ester, methyl jasmonate, operate by initiating signal cascades that result in widespread modification of gene expression patterns affecting hundreds of genes involved in both development and stress responses in plants (Farmer & Ryan, 1990; Vijayan et al., 1998; Kessler, Halitschke & Baldwin, 2004; Reymond et al., 2004; Mandaokar et al., 2006; Thines et al., 2007). In healthy, unwounded plant tissues, jasmonic acid regulates carbon partitioning, mechanotransduction, senescence, and reproductive development (Xie et al., 1998; Xiao et al., 2004; Browse, 2005, Mandaokar et al., 2006). In response to ultraviolet radiation and other abiotic stresses, jasmonic acid regulates damage repair and the plant’s physiological response (Conconi et al., 1996). In response to insect herbivory and pathogen damage, jasmonic acid induces morphological and chemical changes in defense (Farmer & Ryan, 1990; Vijayan et al., 1998; Thaler, 1999; Thaler et al., 2001; Kessler, Halitschke & Baldwin, 2004). We expect jasmonic acid signaling cascades that shape the foliar metabolome to comprise a significant source of intraspecific chemical variation within a tropical forest plant community, though its magnitude relative to interspecific variation remains unclear.

Plant metabolomes also change greatly over the ontogeny of a leaf. Ontogenetic variation in the metabolome is driven in part by changes in the risk of herbivory over the lifetime of a leaf. In tropical forests, leaves experience 70% of lifetime herbivore damage during the relatively brief window during which the leaves develops and expands and before cell walls harden (Coley & Barone, 1996). To compensate for physical vulnerability, young leaves exhibit higher concentrations of chemical defenses such as phenols, tannins and other secondary metabolites (Coley, 1983). Once mature, leaves are defended against most insect herbivores by lignin, cellulose, and physical toughness. Some herbivores, however, are capable of breaching the physical defenses of mature leaves. Hence, we expect fully mature leaves to exhibit qualitatively different metabolomes from those of young, expanding leaves. In addition, we expect the metabolomes of mature leaves to exhibit greater inducible chemical variation, as the jasmonic acid response in mature leaves is likely to signal chemical defenses (Bixenmann, Coley & Kursar, 2011) that cost little to the plant in the absence of herbivory, but that can be activated when a mature-leaf specialist happens upon the leaf. On the other hand, young leaves may exhibit jasmonic acid-inducible variation comparable to mature leaves, despite their physical vulnerability, if the risk of herbivory varies sufficiently for the cost of constitutive chemical foliar defense to be better allocated to growth and reproduction until and unless herbivory occurs (Zangerl & Rutledge, 1996).

Understanding the effect that inducible and ontogenetic variation has on chemical differences between species is particularly important in species-rich communities such as tropical forests, where host-specific biotic interactions may play a fundamental role in stabilizing species coexistence among plants (Wright, 2002). Competitive interactions between conspecific plants must have a greater negative effect on fitness than those between species for stable species coexistence (Chesson & Kuang, 2008). The plant species attacked by a given herbivore or pathogen out of the potential host plants in a community may be strongly influenced by species differences in the diverse secondary metabolites that make up a significant portion of the plant metabolome (Barrett & Heil, 2012). Hence, if variation in foliar metabolomes is sufficient to stabilize species coexistence, jasmonic acid-inducible and ontogenetic variation within species must be small relative to interspecific variation. Furthermore, a substantial proportion of the local species richness of many tropical forests is comprised of a small number of exceptionally species-rich tree genera (Gentry, 1982). High local species richness of congeneric plants poses a challenge to our understanding of diversity maintenance in tropical forests because closely related species are likely to share many aspects of the niche (Webb et al., 2002). For chemically mediated interactions to sustain species coexistence among congeneric species in diverse tree genera, chemical variation must be greater interspecifically than intraspecifically.

Here, we take advantage of recently developed methods to obtain and assemble mass spectra (MS) into molecular networks that quantify the chemical structural similarity of compounds (Wang et al., 2016). Molecular networks make it possible to quantify chemical similarities between samples even though few compounds are unambiguously identified, which is essential in chemically diverse and understudied tropical forests (Sedio, 2017). Sedio et al. (2017) found previously that intraspecific chemical variation due to leaf ontogeny, light environment, and precipitation seasonality was much less than interspecific variation among congeneric species for seven tropical tree species. Here, we examine the effects of jasmonic acid induction, leaf ontogeny, and their interaction on foliar metabolomes in four species that represent four of the most species-rich woody plant genera throughout the Neotropics: *Inga cocleensis* (Fabaceae, Mimosoideae), *Piper cordulatum* (Piperaceae), *Protium panamense* (Burseraceae), and *Psychotria acuminata* (Rubiaceae). We also compare inducible and ontogenetic chemical variation within these species to chemical variation between these species and congeneric species of *Inga*, *Piper*, *Protium*, and *Psychotria*, respectively, with which they co-occur in the Barro Colorado Nature Monument (BCNM), Panama.

## Materials and Methods

### Study site, focal species and genera

The BCNM, Panama (9°9′N, 79°51′W) supports tropical moist forest with 2,600 mm average annual rainfall and a four-month dry season. We sampled 49 species in four genera, including *Inga* (15 species), *Piper* (11 species), *Protium* (3 species), and *Psychotria* (20 species). Molecular phylogenies suggest that *Protium* and *Psychotria* are each paraphyletic with respect to related taxa, forming monophyletic clades only if *Tetragastris* (Fine, Zapata & Daly, 2014) and *Palicourea* (Nepokroeff, Bremer & Sytsma, 1999) are included, respectively. Hence, we include *Tetragastris panamensis* among the *Protium* and *Palicourea guianensis* among the *Psychotria.* One locally abundant species from each genus was chosen for experimental induction of chemical defense using jasmonic acid: *Inga cocleensis*, *Piper cordulatum*, *Protium panamense*, and *Psychotria acuminata*. The species represented by these four genera together comprise 19% of the 409 species—and four of the eight most species-rich genera—of trees and shrubs recorded on Barro Colorado Island (BCI) in the BCNM (Foster & Hubbell, 1990).

### Leaf collection and jasmonic acid treatment

Jasmonic acid treatment is an effective means to induce the expression or modification of anti-herbivore defenses in plants, whereas mechanical damage alone is not effective (Pontoppidan et al., 2005). We followed a jasmonic acid treatment optimized by Thaler et al. (1996) to induce the metabolic cascade produced in response to true herbivory in tomato (*Solanum lycopersicum*). However, the metabolic effects of the Thaler et al. (1996) jasmonic acid treatment in four hitherto unstudied tropical forest tree species may differ from that elicited by insect herbivores or pathogens in four very distantly related plant families. Hence, differences between jasmonic acid and control treatments must be interpreted as metabolomic differences, and not strictly differences in chemical defenses.

We selected at least eight individuals <1.3 m in height of each of the four focal species for treatment with jasmonic acid or a control solution. The treatment was prepared by dissolving 210.27 mg jasmonic acid in 0.125 mL acetone, then bringing the total volume to one L with distilled water to yield a one mmol/L solution. The control solution was prepared by diluting 0.125 mL acetone to one L with distilled water. Individual plants were sprayed with either jasmonic acid or control solution until all leaves were visibly wet in July 2014, following Thaler et al. (1996). Thaler (1999) found that significant effects of jasmonic acid treatment persisted for 3 weeks. Hence, after 2 weeks, we harvested at least four young leaves, as well as at least four leaves that had matured during the 2013 rainy season (following Thaler, 1999) from plants showing no visible herbivore damage. Young leaves were defined as leaves recently flushed, between 50% and 95% fully expanded, but not yet lignified. The experiment was conducted on the Gigante Peninsula, a mainland peninsula adjacent to BCI in the BCNM. During this same period, young, expanding leaves of one individual of each of 45 congeneric species were collected in the shaded forest understory on BCI, including 15 *Inga*, 11 *Piper*, 3 *Protium*, and 20 *Psychotria*. A total of 102 leaf samples were collected on ice and stored at −80 °C within 1 h of collection.

### Liquid chromatography-tandem mass spectrometry (LC-MS/MS)

The methods we employed for chemical extractions and LC-MS/MS analyses follow those reported by Sedio et al. (2017), Sedio, Boya & Rojas Echeverri (2018) and Sedio et al. (2018). We homogenized 100 mg of frozen leaf tissue in a ball mill (Qiagen TissueLyser, Hilden, Germany) and extracted the homogenate with 700 µL 90:10 methanol:water pH 5 for 10 min. The solution was then centrifuged, the supernatant was isolated, and the extraction was repeated on the remaining sample. All solvents, filters, and materials used were UHPLC-MS-grade, following Sedio, Boya & Rojas Echeverri (2018).

Samples were analyzed using reverse phase ultra high-performance liquid chromatography (UHPLC, Agilent Technologies, Santa Clara, CA) with a flow rate of 0.5 mL/min at 25 °C. We developed a 37 min solvent gradient, including a 25 min gradient from 5% to 100% acetonitrile followed by 8 min of isocratic 100% acetonitrile using a Kinetex C18 UHPLC column with 100 mm length, 2.1 mm internal diameter and 1.7µm particle size (Phenomenex, Torrance, CA) to separate compounds characterized by a wide range of hydrophobicity. Both solvents included 0.1% formic acid to facilitate protonation. Liquid chromatographic separation was followed by mass spectrometry detection using electrospray ionization (ESI) in positive mode on a micrOTOF-QIII quadrupole time-of-flight mass spectrometer (Bruker Daltonics, Fremont, CA, USA) with low pictogram sensitivity. Collision energy, acquisition time, and other parameters were optimized so as to detect and fragment molecules representing as wide a range in the mass to charge ratio (m/z) of the parent compound as possible, from ca. 150 m/z to over 1,600 m/z. We used positive mode electrospray ionization to facilitate matching of our spectra using GNPS (Wang et al., 2016) to phytochemical libraries for which the available data was generated in positive mode (e.g., ReSpect; Sawada et al., 2012). Extractions performed without tissue (‘blanks’) were analyzed so as to remove compounds derived from the laboratory environment, and after every five sample runs, 10 min of isocratic acetonitrile was run and the resulting data treated as a blank to remove strongly lipophilic compounds that may have remained in the column after the experimental gradient. Reserpine was used as a lock mass calibrant to control for instrument fluctuations in mass.

Resulting MS/MS spectra of fragmented molecules were clustered using MSCluster, which groups spectra determined to be identical into consensus spectra that represent a single unique compound (Frank et al., 2008). We refer to these consensus spectra as ‘compounds’ throughout. Every pair of compounds was then compared by calculating the cosine of the angle between the vectors that comprise the mass to charge ratio (m/z) of their constituent fragments (Wang et al., 2016; Sedio, Boya & Rojas Echeverri, 2018). Links between molecules with a cosine match score of at least 0.6 were retained in the network. Compounds were identified by comparing MS/MS spectra to spectra in online repositories using the Global Natural Products Social (GNPS) Molecular Networking tool (http://gnps.ucsd.edu; Wang et al., 2016) as described by Sedio, Boya & Rojas Echeverri (2018). The final network is publically available on GNPS (http://gnps.ucsd.edu/ProteoSAFe/status.jsp?task=6d2ad31f795d4975b3d22675b4d24cac). Raw spectra were deposited on GNPS as a MassIVE dataset (massive.ucsd.edu/MSV000084275/; doi:10.25345/C5XT05).

### Gas chromatography-mass spectrometry (GC-MS)

The LC-MS/MS protocol does not capture volatile compounds, so we also performed GC-MS. For GC-MS analysis, we transferred 500 mg of thawed leaf tissue to a four mL vial. The ionic strength was adjusted to 20% with sodium chloride (NaCl). The vial was sealed and the SPME extraction was performed with a DVD/CAR/PDMS fiber (Supelco, Bellefonte, PA, USA). The sample was stirred at 1,200 rpm continuously during the experiment. All extractions were done at 60 °C during 15 min after 20 min incubation. The leaf tissue extracts were analyzed by GC-MS (GC-MS, Agilent Technologies, Santa Clara, CA) using a HP-5MS capillary column, 30 m length, 0.25 mm i.d. and a 0.25 µm phase thickness (Agilent Technologies, Palo Alto, CA, USA). Splitless injection was used. The initial oven temperature was set to 50 °C for 3 min. The temperature was increased to 200 °C at 6 °C min^−1^, and finally up to 280 °C at 10 °C min^−1^, where it was held for 6 min. Transfer line and ion source temperatures were 280 °C and 250 °C respectively. The detection was carried out in EI mode (ionization energy, 70 eV; transfer line temperature, 280 °C; ion source temperature 250 °C). The acquisition was made in SCAN mode (40–400 m/z). The compounds were identified by calculation of Kovats retention indices, which was then compared with the literature; by comparison of the compound mass spectra obtained with that found in the registry of NIST Mass Spectral Data with Structures (Wiley 7th edition, USA); and by the use of pure standards when available. Compound concentrations were quantified by integrating the GC trace.

Molecular networking requires a comparison of the fragmentation patterns of unique molecules provided by MS/MS spectra. Hence, it was not possible to include GC-MS data in molecular networks. GC-MS was not performed on young leaves of *Inga cocleensis* or *Psychotria acuminata*, nor was it applied to congeneric species related to the four focal tree species. As a result, our conclusions regarding inducible variation in young leaves and ontogenetic variation with respect to volatile organic compounds were limited to *Piper cordulatum* and *Protium panamense*, and we were unable to assess interspecific variation in volatiles among congeneric species.

### Chemical structural and compositional similarity (CSCS)

Sedio et al. (2017) developed a chemical structural-compositional similarity (CSCS) metric that weights the structural similarity of every pair of compounds in a network by their relative ion intensity in two samples. Conventional methods of calculating similarity in ecology, such as Bray–Curtis similarity, consider the compositional similarity of compounds, weighted by concentration or ion intensity, while ignoring structural relationships between molecules. In contrast, CSCS accounts for the presence and concentration of identical compounds, as well as distinct but structurally similar compounds that are not shared between samples. A simple example illustrates the implications. Compounds *x* and *y* are structurally similar, species *A* contains compound *x* but not *y*, and species *B* contains *y* but not *x*. In this example, compounds *x* and *y* make no contribution to Bray–Curtis similarity, but make a positive contribution to CSCS.

We calculated CSCS for nonvolatile compounds and Bray–Curtis similarity for both nonvolatile and volatile compounds for every pair of samples (Sedio, Boya & Rojas Echeverri, 2018). Bray–Curtis similarity was calculated using the R package ‘vegan’ (Oksanen et al., 2009). Given 102 samples, there are $ \left( {102\atop 2} \right) =$ 5,151 pairs of samples. Each sample is characterized by its species, leaf age, and jasmonic acid treatment. We then calculated chemical similarities for pairs of samples that differed with respect to just one of these factors and measured the difference between within-factor similarity and between-factor similarity, which we reported as ΔCSCS and ΔBC, respectively.

A permutation test is required to evaluate the significance of differences between treatments because we consider all pairwise combinations of samples. We therefore randomized the assignment of factors to samples and calculated randomized distributions for each difference between within-factor and between-factor similarity. If the observed difference was greater than 95% of the randomized differences, the variable affected chemical similarity significantly. A significant permutation test with respect to Bray–Curtis similarity indicates that distinct compounds are found in samples that differ with respect to a categorical variable or treatment, but does not rule out that those compounds may be structurally similar. A significant permutation test with respect to CSCS similarity indicates that distinct and structurally unrelated compounds are found in samples that differ with respect to a categorical variable or treatment. We performed permutation tests on a single variable while controlling for variation in the other variable by permuting sample pairs that comprise ‘within’- or ‘between’-treatment pairs for the first variable while rejecting any permuted pair that comprised a ‘between’-treatment pair for the second variable. We examined the effect of each variable on all species considered together by combining *p*-values using the weighted-*Z* method (Whitlock, 2005).

### Quantitative variation: total ion intensity

To quantify variation in plant investment in metabolites, we measured total ion intensity of metabolites for each LC-MS sample. Ion intensity is an imperfect measure of concentration. Variation in ion intensity can be influenced by many factors including variation in solubility and ionization efficiency among compounds, and the dependence of both of these factors on the makeup of the molecular community in which a particular compound occurs. However, when ion intensity is summed over tens to hundreds of compounds, differences in the effectiveness with which individual compounds are ionized are likely to offset one another and the remaining variation in total, summed ion intensity is likely to be driven by variation in concentration rather than differences in ionization among molecules. Furthermore, if the difference in ion composition between samples is small, variation in total ion intensity is likely to be driven by concentration.

## Results

**Figure 1:**
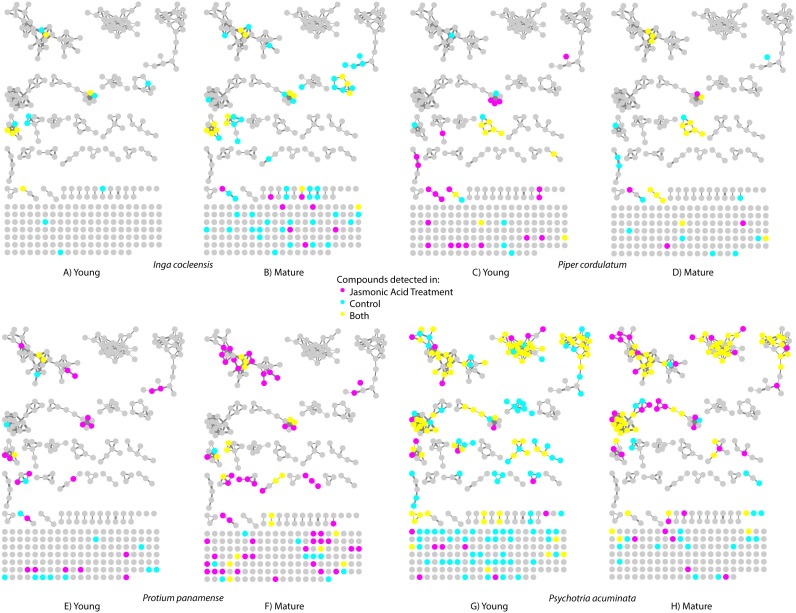
Molecular network indicating the incidence of small molecules in jasmonic acid-treated leaves (magenta), control leaves (cyan), and both (yellow) for four species. Nodes represent compounds (e.g., caffeine); links between nodes indicate molecular structural similarity between compounds (e.g., caffeine and theobromine). (A) Young and (B) mature leaves of *Inga cocleensis*, (C) young and (D) mature leaves of *Piper cordulatum*, (E) young and (F) mature leaves of *Protium panamense*, and (G) young and (H) mature leaves of *Psychotria acuminata*.

The LC-MS/MS protocol isolated 1,302 putative compounds (see Methods for a definition of compound). Networks of compounds linked by a cosine match score of ≥ 0.6 ranged in size from 2 to 749 compounds (Fig. 1). The remaining 498 compounds had cosine similarity scores <0.6 with every other compound found in the 102 leaf samples. The GC-MS protocol identified an additional 158 volatile compounds. Compounds identified using MS/MS matches to GNPS spectral libraries (LC-MS/MS data) and Kovats retention indices (GC-MS data) are shown in Tables S1 and S2, respectively. Library matches include benzoquinones, flavonoids, indole alkaloids, quinolones, piperidines, and terpenoids, classes of plant secondary compounds known to include anti-herbivore defenses (Tables S1–S2, Figs. S1–S3). Library matches also include primary metabolites and their degradation products, including the porphyrins pheophytin and pheophorbide A that result from chlorophyll degradation (Tables S1–S2).

### Jasmonic acid induction

We detected distinct compounds in jasmonic acid-treated and control leaves in both young and fully mature leaves for all four species (Fig. 1). Table S1 presents compounds detected in jasmonic acid-induced leaves identified by matching MS/MS spectra to MS libraries.

**Figure 2:**
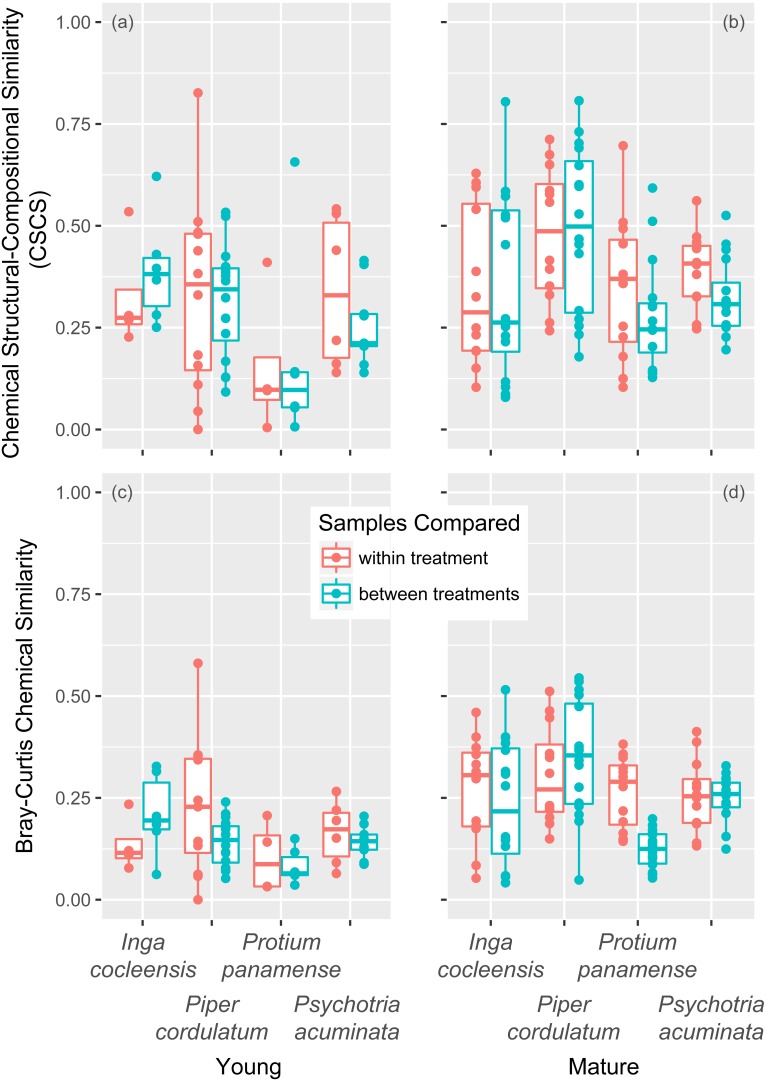
Chemical similarity within and between jasmonic acid treatment in young and mature leaves. Chemical similarity between pairs of leaf samples is represented by chemical structural compositional similarity (CSCS) for young (A) and mature (B) leaves and by Bray–Curtis chemical similarity for young (C) and mature (D) leaves. Each point represents the similarity between two leaf samples. Boxplots indicate the median and the first and third quartiles of chemical similarity for pairwise sample combinations.

Chemical structural compositional similarity (CSCS) rarely differed significantly with jasmonic acid treatment. For young leaves, treatment and control differed significantly for zero species (Table 1A). For mature leaves, treatment and control differed significantly for one of the four species (*Psychotria acuminata*; ΔCSCS = 0.07, *p* = 0.029, Table 1B). CSCS was greater within than between the jasmonic acid and control treatments (i.e., ΔCSCS >0) for two species for both young and mature leaves (Tables 1A and 1B, Figs. 2A and 2B). The jasmonic acid treatment did not have a significant effect on either young or mature leaves when all species were considered together by combining *p*-values with the weighted-*Z* method, though between-treatment pairs tended to have lower CSCS similarity than within-treatment pairs in mature leaves (Table 1B).

**Table 1:** The effect of jasmonic acid treatment on chemical structural compositional similarity (CSCS) Bray–Curtis similarity, and total ion intensity. Nonvolatile compounds were detected using LC-MS/MS; volatiles were detected using GC-MS. For CSCS and BrayCurtis similarity, significance refers to the proportion of 999 differences in chemical similarity between within-species and between-species sample pairs calculated for permuted datasets that were larger than the observed difference. For total ion intensity, we tested the hypothesis that total metabolomic ion intensity was greater in jasmonic acid-treated than in control leaves using a one-sided Wilcoxon rank sum test.

A. Nonvolatile compounds, young leaves												
	CSCS				Bray–Curtis similarity				Total Ion Intensity			
Species	Within	Btwn	Δ	*p*	Within	Btwn	Δ	*p*	JA	Ctl	Δ	*p*
*I. cocleensis*	0.33	0.39	−0.06	1.000	0.14	0.21	−0.07	0.800	4.17	4.70	−0.53	0.600
*Pi. cordulatum*	0.33	0.31	0.01	0.429	**0.23**	**0.14**	**0.09**	**0.029**	4.55	2.09	2.45	0.243
*Pr. panamense*	0.15	0.18	−0.02	0.500	0.10	0.08	0.02	0.400	3.58	2.85	0.73	0.600
*Ps. acuminata*	0.34	0.26	0.08	0.100	0.16	0.14	0.02	0.300	19.30	67.85	−48.55	0.800
Combined *p*^#^				0.306				**0.046**				0.565

B. Nonvolatile compounds, mature leaves												
	CSCS				Bray–Curtis similarity				Total Ion Intensity			
Species	Within	Btwn	Δ	*p*	Within	Btwn	Δ	*p*	JA	Ctl	Δ	*p*
*I. cocleensis*	0.35	0.35	0.00	0.457	0.27	0.24	0.03	0.114	6.28	12.14	−5.85	0.900
*Pi. cordulatum*	0.48	0.49	−0.01	0.571	0.30	0.35	−0.04	0.971	2.07	2.88	−0.81	0.900
*Pr. panamense*	0.35	0.28	0.08	0.086	**0.26**	**0.12**	**0.14**	**0.026**	14.31	1.81	12.50	**0.014**
*Ps. acuminata*	**0.39**	**0.32**	**0.07**	**0.029**	0.26	0.25	0.01	0.371	10.52	8.18	2.34	0.657
Combined *p*^#^				0.055				0.216				0.650

C. Volatile compounds, Bray–Curtis similarity								
	Young leaves				Mature leaves			
Species	Within	Btwn	Δ	*p*	Within	Btwn	Δ	*p*
*I. cocleensis*					0.39	0.33	0.05	0.100
*Pi. cordulatum*	0.46	0.50	−0.04	0.914	0.43	0.37	0.06	0.086
*Pr. panamense*	0.44	0.27	0.17	0.333	0.34	0.38	−0.04	0.886
*Ps. acuminata*					0.52	0.49	0.04	0.171
Combined *p*^#^				0.908				0.200

**Notes.**

# Combined *p*-values indicate the significance of the effect of leaf age over all species using the weighted *Z*-method.

The jasmonic acid treatment had a stronger effect on Bray–Curtis chemical similarity. For young leaves, treatment and control differed significantly for one of four species (*Piper cordulatum*; Table 1A). For mature leaves, treatment and control also differed for one of four species (*Protium panamense*; Table 1B). Within-treatment Bray–Curtis similarity was greater than between-treatment similarity in six of eight comparisons over both young and mature leaves (Tables 1A and 1B, Figs. 2C and 2D). Jasmonic acid treatment had a significant effect on young leaves when all species were considered together by combining *p*-values with the weighted-*Z* method (Table 1A).

For volatile compounds, the jasmonic acid treatment had no significant effects on Bray–Curtis similarity for any species in either young or mature leaves (Table 1C). Within-treatment Bray–Curtis similarity was greater than between-treatment similarity in mature leaves in three of four species; however, the effect was not significant when all species were considered together using the weighted-*Z* method (Table 1C).

Total ion intensity rarely differed significantly with jasmonic acid treatment (Tables 1A, 1B). For young leaves, treatment and control differed significantly for zero species (Table 1A). For mature leaves, treatment and control differed significantly for one of the four species (*Protium panamense*; Table 1B). Jasmonic acid treatment did not have a significant effect when all species were considered together by combining *p-* values (Tables 1A, 1B). However, total ion intensity was greater in treatment than in control leaves for every ontogeny-species combination that exhibited a significant effect of jasmonic acid treatment on either CSCS or Bray–Curtis similarity (Tables 1A, 1B).

### Leaf ontogeny

**Figure 3:**
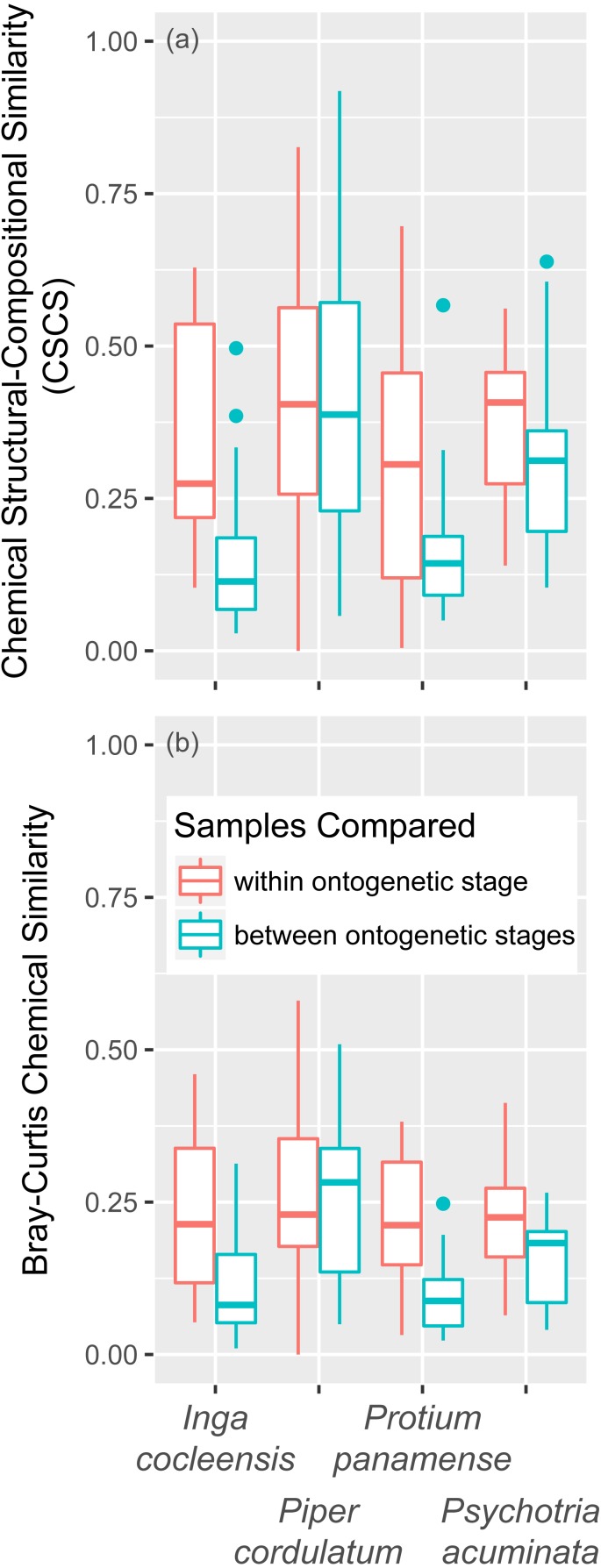
Chemical similarity within and between leaf ontogenetic stages. Chemical similarity between pairs of leaf samples is represented by chemical structural compositional similarity (CSCS; A) and Bray–Curtis similarity (B). Boxplots indicate the median and the first and third quartiles of chemical similarity for pairwise sample combinations.

**Figure 4:**
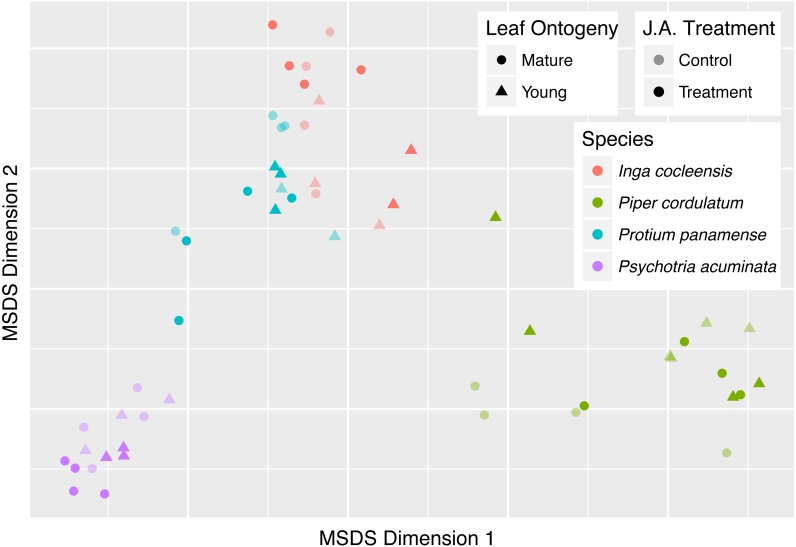
Classical multidimensional scaling (MSDS) of pairwise CSCS similarity among all pairs of leaf samples in four focal species. Young and mature leaves are indicated by triangles and circles, respectively. Jasmonic acid and control treatment samples are indicated by bold and pale points, respectively. Species is indicated by color.

The effect of leaf age on nonvolatile chemical similarity, controlling for jasmonic acid treatment, was strongly significant in three of four species, whether measured in terms of CSCS or Bray–Curtis similarity. Within-leaf age chemical similarity was greater than between leaf-age chemical similarity in seven of eight comparisons (Table 2, Fig. 3). Leaf age had a significant effect on both CSCS and Bray–Curtis similarity when all species were considered together by combining *p*-values using the weighted-*Z* method (Tables 2A, 2B). The combined effects of jasmonic acid treatment and leaf ontogeny can be seen for each focal species in Fig. 4.

**Table 2:** The effect of leaf age on chemical structural compositional similarity (CSCS) and Bray–Curtis similarity, controlling for jasmonic acid treatment, and on total ion intensity in control leaves. Nonvolatile compounds were detected using LC-MS/MS; volatile compounds were detected using GC-MS. Significance refers to the proportion of 999 differences in chemical similarity between within-species and between-species sample pairs calculated for permuted datasets that were larger than the observed difference. For total ion intensity, we tested the hypothesis that total metabolomic ion intensity was greater in young than in mature leaves using a one-sided Wilcoxon rank sum test.

A. Nonvolatile compounds												
	CSCS				Bray–Curtis similarity				Total Ion Intensity			
Species	Within	Btwn	Δ	*p*	Within	Btwn	Δ	*p*	Young	Mature	Δ	*p*
*I. cocleensis*	**0.34**	**0.15**	**0.19**	**0.010**	**0.24**	**0.11**	**0.13**	**0.010**	4.70	12.14	−7.44	0.943
*Pi. cordulatum*	0.40	0.41	−0.00	0.471	0.27	0.25	0.01	0.300	2.09	2.88	−0.788	0.9
*Pr. panamense*	**0.30**	**0.17**	**0.13**	**0.017**	**0.22**	**0.10**	**0.13**	**0.009**	2.85	1.81	1.04	0.135
*Ps. acuminata*	**0.38**	**0.30**	**0.08**	**0.013**	**0.23**	**0.16**	**0.07**	**0.004**	67.85	8.18	59.67	0.057
Combined *p*^#^				<**0.001**				<**0.001**				0.602

B. Volatile compounds				
	Bray–Curtis similarity			
*I. cocleensis*				
*Pi. cordulatum*	**0.45**	**0.13**	**0.32**	**0.001**
*Pr. panamense*	0.35	0.34	0.02	0.371
*Ps. acuminata*				
Combined *p*^#^				**0.007**

**Notes.**

# Combined *p*-values indicate the significance of the effect of leaf age over all species using the weighted *Z*-method.

For volatile compounds, the effect of leaf age on Bray–Curtis chemical similarity, controlling for jasmonic acid treatment, was significant for one of two species (Table 2C). The only species for which leaf age did not significantly affect nonvolatile chemistry, *Piper cordulatum*, exhibited a significant effect of leaf age on Bray–Curtis chemical similarity of volatile compounds (Table 2C).

The effect of leaf age on total ion intensity was not significant for any species (Table 2A). Mean total ion intensity was greater in young than in mature leaves for two species. The mean difference was large but not significant for *Psychotria acuminata*. Leaf age did not have a significant effect on total ion intensity when all species were considered together by combining *p*-values (Table 2A).

### Interspecific chemical variation among congeneric species

**Figure 5:**
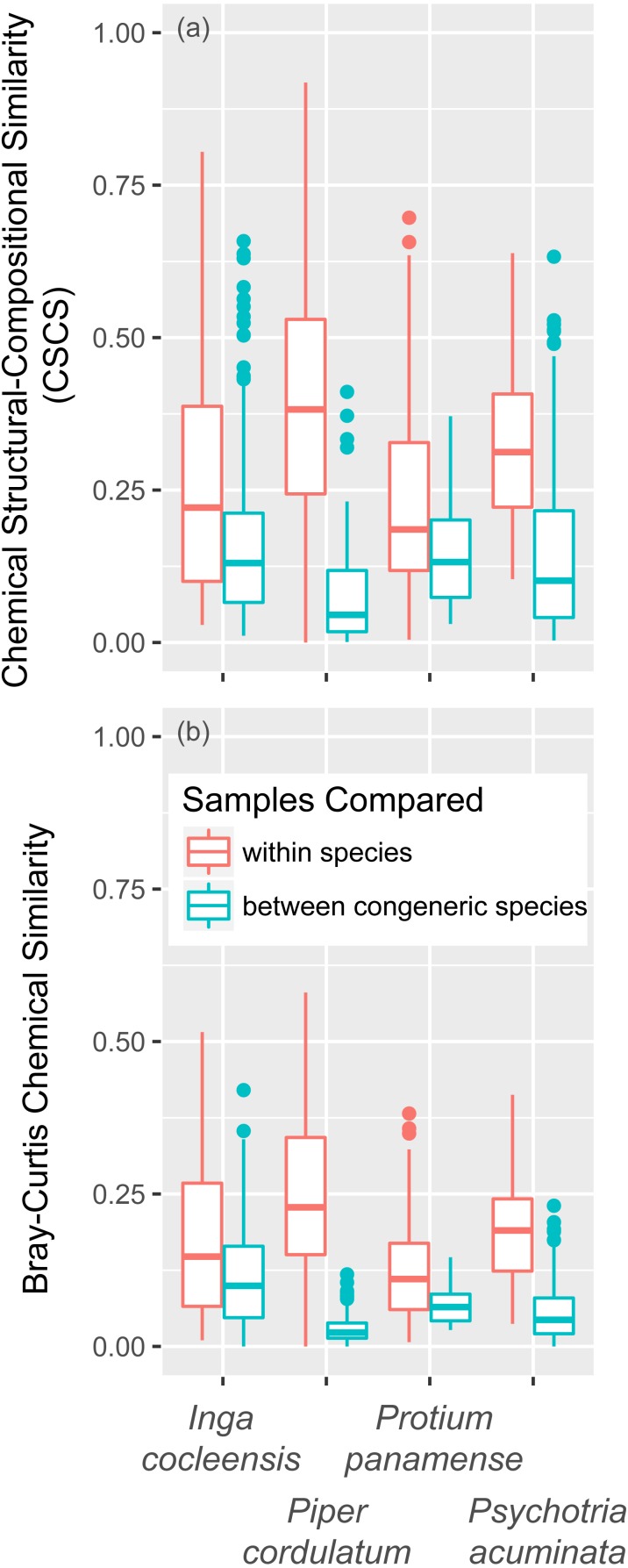
Chemical similarity within species and between congeneric species. Chemical similarity between pairs of leaf samples is represented by chemical structural-compositional similarity (CSCS; A) and Bray–Curtis similarity (B). Within-species comparisons include every pairwise combination of samples within each focal species (*I. cocleensis*, *P. cordulatum*, *P. panamense*, and *P. acuminata*), including within- and between-jasmonic acid treatment and leaf age pairs. Between-species sample pairs include every leaf sample of the four focal species paired with young, untreated leaf samples of each congeneric species, including 14, 10, 2 and 19 species of *Inga*, *Piper*, *Protium* and *Psychotria*, respectively. Boxplots indicate the median and the first and third quartiles of chemical similarity for pairwise sample combinations.

Focal species of *Inga*, *Piper*, and *Psychotria* were chemically different from congeneric species, whether measured in terms of CSCS or Bray–Curtis similarity (Table 3). *P. panamense* differed significantly from other *Protium* with respect to Bray–Curtis but not CSCS chemical similarity. CSCS and Bray–Curtis similarity were much greater within than between species, even when conspecific comparisons were made with leaves that differed in jasmonic acid treatment and leaf age (Fig. 5). This was true for all four genera (Fig. 5).

**Table 3:** Chemical Structural Compositional Similarity (CSCS) and Bray–Curtis (BC) similarity within and between congeneric species. Within-species sample pairs included both jasmonic acid-treated and control and both young and mature leaves for the four focal species (*Inga cocleensis*, *Piper cordulatum*, *Protium panamense*, and *Psychotria acuminata*). Between-species sample pairs include every leaf of each focal species paired with a young, control leaf of a congeneric species, including 14, 10, 2 and 19 species of Inga, Piper, Protium, and Psychotria, respectively. Significance refers to the proportion of 999 differences in chemical similarity between within-species and between-species sample pairs calculated for permuted datasets that were larger than the observed difference.

Genus	Within	Between	Δ	Significance
(A) CSCS similarity				
*Inga*	0.26	0.17	0.09	**0.006**
*Piper*	0.40	0.07	0.32	<**0.001**
*Protium*	0.23	0.15	0.08	0.105
*Psychotria*	0.32	0.15	0.17	<**0.001**
(B) Bray–Curtis (BC) similarity				
*Inga*	0.18	0.11	0.07	**0.004**
*Piper*	0.25	0.03	0.22	<**0.001**
*Protium*	0.13	0.07	0.06	**0.048**
*Psychotria*	0.19	0.05	0.13	<**0.001**

## Discussion

The effect of treatment with the defense-inducing plant hormone jasmonic acid varied by species, by leaf ontogenetic stage, and by the metric used to measure the effect in ways that suggest that inducible chemical defenses may not be universal in tropical trees (Bixenmann et al., 2016) and may vary widely among species. In contrast. young, expanding leaves consistently differed in their chemical composition compared to fully mature leaves in all four focal species and interspecific chemical variation was much greater than variation within species for all four genera, even when within-species comparisons included leaves that differed in age and jasmonic acid treatment. Our results support the optimal defense hypothesis that young, physically vulnerable leaves should exhibit a distinct composition of secondary metabolites compared to matures leaves (Coley, 1983; Wiggins et al., 2016) and suggest that despite ontogenetic and inducible variation within species, chemical differences among congeneric species may be sufficient to partition niche space with respect to chemical defense.

### Inducible chemical variation

Bray–Curtis similarity treats compounds with distinct structures as independent, and non-zero contributions to bray–curtis similarity only occur when samples share a compound (Oksanen et al., 2009). In contrast, CSCS reflects not only concentrations of shared compounds, but also concentrations of distinct but structurally similar compounds. If CSCS is high and Bray–Curtis similarity is low, the samples share distinct but structurally similar compounds.

Young leaves showed a significant chemical response to jasmonic acid in terms of Bray–Curtis chemical similarity but not in terms of CSCS (Table 1A, Fig. 2). This observation suggests that jasmonic acid-induced young leaves express compounds that are different from, yet structurally related to, compounds expressed in uninduced control leaves. The metabolomic effect of jasmonic acid treatment may have a stronger effect on bray–curtis than on CSCS chemical similarity if the affected metabolic pathways take advantage of structurally similar precursors present in uninduced leaves. Mature leaves, meanwhile, exhibited a marginally significant effect of jasmonic acid induction on CSCS (Table 1B), which suggests that jasmonic acid may activate metabolic pathways that are not otherwise active in the mature leaf.

Optimal defense theory predicts that constitutive, rather than inducible, defenses should be favored in environments in which the risk of herbivory is consistently high, such as tropical forests (Coley, 1983; Zangerl & Rutledge, 1996). Few studies have examined inducible chemical defenses in tropical trees, but the lack of widespread evidence of inducible variation in the metabolome in our focal species lends support to the conclusions of Bixenmann et al. (2016) that inducible variation does not play a large role in the defensive strategies employed by tropical rainforest trees.

### Leaf ontogenetic chemical variation

Coley (1983) proposed that young leaves rely on chemical defenses because they cannot be protected by physical toughness. Our results support the prediction that young and mature leaves differ qualitatively in their small-molecule metabolomes (Coley, 1983; Table 2; Fig. 3; Coley & Barone, 1996; Wiggins et al., 2016). Leaf ontogenetic stage demonstrated a significant effect on both CSCS and bray–curtis chemical similarity (Table 2, Fig. 3). This suggests that compounds expressed in expanding, unlignified leaves are not structurally similar to those found in fully mature leaves, perhaps because distinct metabolic pathways are expressed in vulnerable, growing leaves and fully hardened, mature leaves in these species, including those involved in defense (Wiggins et al., 2016).

Large differences in the metabolomic makeup of young and matures leaves may be enhanced by ontogenetic variation in jasmonic acid-inducible secondary metabolites involved in anti-herbivore defense. Young leaves experience much greater rates of herbivore damage than fully mature leaves (Coley, 1983). The optimal defense hypothesis predicts that young leaves should express chemical defenses constitutively because of the high threat of herbivory and physical vulnerability (Stamp, 2003; Bixenmann, Coley & Kursar, 2011). Mature leaves, protected from some herbivores by physical toughness, are more likely to exhibit inducible chemical defenses, useful when an herbivore tolerant of the toughness of mature leaves attacks but otherwise unneeded. Our results indeed indicate large differences in metabolomic composition between young and mature leaves in all four species (Table 2, Figs. 1 and 3). However, in contrast with expectations, constitutive investment in metabolites was not quantitatively greater in young than in mature leaves, with the possible exception of *Psychotria acuminata*, for which young leaves exhibited a total ion intensity eight times greater than that of mature leaves (Table 2)*.* Wiggins et al. (2016) found that expanding leaves exhibited much greater investment in secondary metabolites by weight than did mature leaves of six species of *Inga*. The contrast between the results of Wiggins et al. (2016) and those reported here may be attributed to the weakness of total ion intensity as a measure of quantitative investment in chemical defense.

We did not observe a consistent difference in the effect of jasmonic acid treatment on chemical composition between young and mature leaves (Table 1, Fig. 2). Some species, such as *Protium panamense* and *Psychotria acuminata*, may employ inducible chemical defense in mature leaves. These results are consistent with the hypothesis that mature leaves, for which the risk of herbivory is low, should be more likely to express inducible chemical defense (Zangerl & Rutledge, 1996; Bixenmann et al., 2016). However, the variation we observed among our four focal species suggests that inducible variation in expanding leaves is rare in tropical trees (Bixenmann et al., 2016) but that strategies may vary among species.

Insect herbivores exhibit broad phylogenetic signal in feeding guild, defined by the plant tissues on which they feed at each insect developmental stage (Novotny et al., 2010). The large differences we observed in CSCS between young and mature leaves raise the possibility that qualitatively different chemical defenses are expressed over ontogeny to defend against distinct herbivore taxa, which may vary in their sensitivity to particular compounds or even chemical classes (Barrett & Heil, 2012).

### Interspecific chemical variation and implications for enemy-mediated competitive coexistence

Chemical variation within our four focal species due to jasmonic acid induction and leaf ontogeny was much less than the chemical variation between each focal species and congeneric species with which the focal species co-occur within the Barro Colorado Nature Monument in Panama. The high local species richness exhibited by some tropical tree genera may be maintained if congeneric species exploit distinct chemical niches, particularly those defined by defenses against herbivores and pathogens, such that negative interactions mediated by shared pests are greater within-species than between-species (Holt & Lawton, 1994; Chesson & Kuang, 2008; Sedio & Ostling, 2013). Species niche differences necessary for stable coexistence might be obscured if intraspecific inducible and ontogenetic chemical variation were large relative to interspecific variation. Alternatively, inducible and ontogenetic variation may be small relative to species differences. Our results indicate that inducible and ontogenetic variation in the foliar metabolome within species does not obscure chemical differences among congeneric species of *Inga*, *Piper*, *Protium*, and *Psychotria* (Table 2, Fig. 5). The chemical similarity between congeneric species is clearly much less than that between conspecific leaf samples, even when the conspecific leaf samples include different leaf developmental stages and jasmonic acid treatments (Fig. 5). These results suggest that species differences in secondary chemistry are large relative to inducible and ontogenetic chemical variation within species, perhaps sufficiently large to allow congeneric species to partition niche space based on chemical defenses against insects and other plant enemies. Such interspecific chemical variation may be particularly important for defining chemical niche differences among co-occurring congeners in ‘species swarm’ tree genera such as *Inga*, *Piper*, *Protium*, and *Psychotria*, which comprise a major part of the chemical diversity of tropical forests (Sedio et al., 2018).

### Caveats to the interpretation of jasmonic acid-induced variation in foliar metabolomes

The whole-leaf metabolomes considered here likely include small molecules involved in core metabolic and signaling pathways, in addition to secondary metabolites that include anti-herbivore and anti-microbial defenses. Hence, inducible and ontogenetic variation in chemical defenses *per se* may be less than the variation documented here for the more inclusive metabolome. On the other hand, the large differences we observed among congeneric species are likely driven by variation in secondary chemistry, rather than by signaling or core metabolic pathways, as the former is more likely to be under selection for divergence among closely related species (Becerra, 1997; Kursar et al., 2009; Salazar, Jaramillo & Marquis, 2016; Coley, Endara & Kursar, 2018; Salazar et al., 2018; Volf et al., 2018).

The jasmonic acid treatment was performed in the field, without accounting for variation in the age, diameter, or height of individuals, or the edaphic environment or neighborhood species composition of their localities. Individual ontogeny and physiological and biotic stress are all likely to influence the response of plants to jasmonic acid treatment (Conconi et al., 1996; Kessler, Halitschke & Baldwin, 2004), and may increase inducible variation relative to what we observed.

In addition, though the jasmonic acid treatment we employed was designed to be a systemic treatment (Thaler, 1999), it is possible that the different response we observed among young and matures leaves may be due to variation in the effectiveness of the treatment itself among tissues in the plant. If the effect of the treatment was local and not systemic, young leaves may have received a greater dose of jasmonic acid due to greater surface permeability. This possibility could be tested by treating specific plant organs locally and testing for a systemic response in plant organs not directly treated with the signaling compound.

Furthermore, the general methanolic extraction and specific LC-MS conditions (e.g., column, solvents, and ionization mode) favor hydrophilic and nitrogen-containing compounds and may neglect some nonvolatile lipophilic compounds that may contribute to ontogenetic and inducible variation. Such undetected variable and inducible metabolites may include compounds that function in signaling or defense. Additional analyses that utilize alternative solvents and ionization methods may expand the metabolomic range of this approach.

Finally, our conclusions are necessarily limited by the small number of species we considered. However, very few studies have examined inducible chemical variation in tropical trees (Boege, 2004; Bixenmann et al., 2016), especially in tropical moist forests. The four focal species considered here represent four phylogenetically distantly related (Kress et al., 2009) and species-rich genera. Hence, our results comprise phylogenetically independent observations that are relevant to understanding species diversity in tropical forest tree communities.

### Future Directions

Recent developments in the bioinformatics of molecular networks have improved upon the methods we utilized in this study in several key respects. For example, the MS-Cluster algorithm (Frank et al., 2008) for grouping spectra into consensus spectra does not consider differences in LC retention time that typically distinguish structural isomers with identical molecular masses. The GNPS molecular networking tool recently integrated software, such as MZmine 2 (Pluskal et al., 2010), to resolve isomeric compounds, annotate molecular networks with putative chemical formulas, and improve the quantification of variation in ion abundances among samples. Future applications should integrate these methods into molecular networks to improve our ability to identify particular compounds or ‘features’ that distinguish treatments or species while still taking advantage of the capacity of molecular networks to assess structural relatedness and redundancy.

## Conclusions

Plant interactions with other organisms are mediated by chemistry, yet chemistry varies among and within conspecific individual plants. Within-species variation in the foliar metabolome—the suite of small-molecule metabolites found in the leaf—changes during leaf ontogeny and is influenced by the signaling molecule jasmonic acid. Here, we examined how such intraspecific chemical variation compares with the species differences thought to play an important role in niche differentiation, and hence species coexistence, in species-rich plant communities such as tropical forests. We observed significant effects of the jasmonic acid treatment for only three of eight combinations of species and ontogenetic stage evaluated, consistent with the hypothesis that constitutive, rather than inducible, defenses should be favored in environments in which the risk of herbivory is high (Zangerl & Rutledge, 1996). Three of the four species also exhibited large metabolomic differences with leaf ontogenetic stage. The profound effect of leaf ontogenetic stage on the foliar metabolome suggests a qualitative turnover in secondary chemistry with leaf ontogeny. We also quantified foliar metabolomes for 45 congeners of the four focal species. Chemical similarity was much greater within than between species for all four genera, even when within-species comparisons included leaves that differed in age and jasmonic acid treatment. Despite ontogenetic and inducible variation within species, our results suggest that chemical differences among congeneric species may be sufficient to partition niche space with respect to chemical defense.

##  Supplemental Information

10.7717/peerj.7536/supp-1 Table S1 Compounds that matched records in Global Natural Products Social (GNPS) Molecular Networking mass spectra libraries (gnps.ucsd.edu), with cosine scores higher than 0.6 Annotated spectra with cosine scores higher than 0.8 are indicated with an asterisk (*).

10.7717/peerj.7536/supp-2 Table S2 Volatile compounds found in young and mature leaves of each species Volatile compounds were detected using GC-MS and identified by comparing Kovats retention indices to the literature.

10.7717/peerj.7536/supp-3 Supplemental Information 1 Procyanidin B2 HRESIqTOF-MS *m*∕*z* 579.1510 [*M* + *H*]^+^ (calculated for C30H27O12, 575.1497). mMass mirror view of MS/MS from experimental data and GNPS compound CCMSLIB00000081689 (bottom spectrum) that are associated to the structure in the top righ Annotated Spectra for compounds that matched records in Global Natural Products Social (GNPS) Molecular Networking mass spectra libraries with cosine score above 0.8 (http://gnps.ucsd.edu ID=6d2ad31f795d4975b3d22675b4d24cac). Each spectrum collected using a high resolution q-TOF mass spectrometer with an electrospray ionization source (HRESIqTOF-MS) was compared with GNPS database spectra using mirror view with mMass (version 5.5.0), followed by evaluation of the major collision induced dissociation (CID) as described by Demarque et al. (DOI: 10.1039/c5np00073d), and finally molecular formula was calculated using precursor ion mass with Bruker Compass Data Analysis 4.1.

10.7717/peerj.7536/supp-4 Supplemental Information 2 Epicatechin HRESIqTOF-MS *m*∕*z* 291.0876 [*M* + *H*]^+^ (calculated for C_15_H_15_O_6_, 291.0863). mMass mirror view of MS/MS experimental and GNPS compound CCMSLIB00000081479 (bottom spectrum) that are associated to the structure Annotated Spectra for compounds that matched records in Global Natural Products Social (GNPS) Molecular Networking mass spectra libraries with cosine score above 0.8 (http://gnps.ucsd.edu ID=6d2ad31f795d4975b3d22675b4d24cac). Each spectrum collected using a high resolution q-TOF mass spectrometer with an electrospray ionization source (HRESIqTOF-MS) was compared with GNPS database spectra using mirror view with mMass (version 5.5.0), followed by evaluation of the major collision induced dissociation (CID) as described by Demarque et al. (DOI: 10.1039/c5np00073d), and finally molecular formula was calculated using precursor ion mass with Bruker Compass Data Analysis 4.1.

10.7717/peerj.7536/supp-5 Supplemental Information 3 TAXIFOLIN mMass mirror view of MS/MS from experimental data and GNPS Library Spectrum CCMSLIB00000078866 (bottom spectrum) that are associated to the structure in the top right corner Annotated Spectra for compounds that matched records in Global Natural Products Social (GNPS) Molecular Networking mass spectra libraries with cosine score above 0.8 (http://gnps.ucsd.edu ID=6d2ad31f795d4975b3d22675b4d24cac). Each spectrum collected using a high resolution q-TOF mass spectrometer with an electrospray ionization source (HRESIqTOF-MS) was compared with GNPS database spectra using mirror view with mMass (version 5.5.0), followed by evaluation of the major collision induced dissociation (CID) as described by Demarque et al. (DOI: 10.1039/c5np00073d), and finally molecular formula was calculated using precursor ion mass with Bruker Compass Data Analysis 4.1.

## Additional Information and Declarations

### Data Availability

The following information was supplied regarding data availability: The metabolomic data analyzed in the present manuscript is curated and publicly available through the Global Natural Products Social (GNPS) Molecular Networking platform as a molecular network (http://gnps.ucsd.edu/ProteoSAFe/status.jsp?task=6d2ad31f795d4975b3d22675b4d24cac) and as a MassIVE dataset curated by the Center for Computational Mass Spectrometry at the University of California, San Diego (http://massive.ucsd.edu/MSV000084275/; doi:10.25345/C5XT05).
