# Supplementary material for: A comparison of inducible, ontogenetic, and interspecific sources of variation in the foliar metabolome in tropical trees
Source: PeerJ. 2019 Sep 20;7:e7536. doi: 10.7717/peerj.7536 (PMC6756142; doi:10.7717/peerj.7536)
Supplement: Table S1 — Annotated spectra with cosine scores higher than 0.8 are indicated with an asterisk (*). [file peerj-07-7536-s001.docx]

Table S1. Compounds that matched records in Global Natural Products Social (GNPS) Molecular Networking mass spectra libraries (gnps.ucsd.edu), with cosine scores higher than 0.6. Annotated spectra with cosine scores higher than 0.8 are indicated with an asterisk (*).

| Species | Compound Match in GNPS Library | Compound Class |
| --- | --- | --- |
| *I. cocleensis*  Young leaves | Nadifloxacin | Quinolones (pyridoquinoline) |
| *I. cocleensis*  Mature leaves | Erucamide | Fatty acids (Erucic acid) |
|  | Haloperidol (Haldol) | Piperidines |
|  | 11-(4-Hydroxyphenyl)-2,2,6,6-tetramethyl-3,4-dihydro-2H,6H,12H-dipyrano[2,3-f:2',3'-h]chromen-12-one | Isoflavone |
|  | Xylometazoline | Imidazoles |
|  | N-cyclopropyl-3-(2,3,5-trimethyl-7-oxofuro[3,2-g]chromen-6-yl)propanamide | Chromanones |
|  | Nadifloxacin | Quinolones (pyridoquinoline) |
|  | Pheophorbide A | Porphyrins |
|  | Pheophytin | Porphyrins |
|  | Protoporphyrin IX | Porphyrins |
|  | Putative Digalactosyl diacylglycerol (14:0/16:0) | Glycosyldiacylglycerols |
|  | Reserpine | Indole Alkaloids |
| *I. cocleensis*  Young leaves treated with jasmonic acid | None |  |
| *I. cocleensis*  Mature leaves treated with jasmonic acid | Nadifloxacin | Quinolones (pyridoquinoline) |
| *Pi. cordulatum*  Young leaves | Clitorin | Flavonols |
| *Pi. cordulatum*  Mature leaves | Avobenzone (Parsol 1789) | Propiophenones |
|  | Erucamide | Fatty acids (Erucic acid) |
|  | Xylometazoline | Imidazoles |
|  | Pheophorbide A | Porphyrins |
| *Pi. cordulatum*  Young leaves treated with jasmonic acid | Avobenzone (Parsol 1789) | Propiophenones |
|  | Erucamide | Fatty acids (Erucic acid) |
|  | Exemestane | Oxo steroid |
|  | Clitorin | Flavonols |
|  | Xylometazoline | Imidazoles |
|  | N-cyclopropyl-3-(2,3,5-trimethyl-7-oxofuro[3,2-g]chromen-6-yl)propanamide | Chromanones |
|  | Pheophorbide A | Porphyrins |
| *Pi. cordulatum*  Mature leaves treated with jasmonic acid | None | None |
| *Pr. panamense*  Young leaves | None | None |
| *Pr. panamense*  Mature leaves | Quercitrin | Flavonols |
|  | Nadifloxacin | Quinolones (pyridoquinoline) |
|  | Taxifolin* | Flavonols |
| *Pr. panamense*  Young leaves treated with jasmonic acid | Epicatechin* | Flavonoids (Flavans) |
|  | Erucamide | Fatty acids (Erucic acid) |
|  | N-cyclopropyl-3-(2,3,5-trimethyl-7-oxofuro[3,2-g]chromen-6-yl)propanamide | Chromanones |
|  | Quercitrin | Flavonols |
|  | Pheophorbide A | Porphyrins |
|  | Procyanidin B2* | Tannins (Proanthocyanidins) |
|  | trans-5-O-Caffeoylquinic acid* | Tannins (Cinnamates) |
| *Pr. panamense*  Mature leaves treated with jasmonic acid | 1-Palmitoyl-sn-glycero-3-phosphocholine | Lysophosphatidylcholines |
|  | Epicatechin* | Flavonoids (Flavans) |
|  | Erucamide | Fatty acids (Erucic acid) |
|  | N-cyclopropyl-3-(2,3,5-trimethyl-7-oxofuro[3,2-g]chromen-6-yl)propanamide | Chromanones |
|  | Quercitrin | Flavonols |
|  | Nadifloxacin | Quinolones (pyridoquinoline) |
|  | Pheophorbide A | Porphyrins |
|  | Procyanidin B2* | Tannins (Proanthocyanidins) |
|  | Putative Digalactosyl diacylglycerol (14:0/16:0) | Glycosyldiacylglycerols |
|  | Rutin | Flavonoids (Glycosyloxyflavone) |
|  | Taxifolin* | Flavonols |
| *Ps. Acuminate*  Young leaves | 5-epi-ilimaquinone | benzoquinones |
|  | Argatroban | Pipecolic Acids |
|  | Hyperoside | Flavonols |
|  | Benzocaine | Benzoates |
|  | Imidocarb dipropionate | Carbanilides |
|  | Cisapride | Aminobenzoic acids |
|  | Karakoline | Alkaloids |
|  | Matsukaze Lactone | Lactone |
|  | Putative Digalactosyl diacylglycerol (14:0/16:0) | Glycosyldiacylglycerols |
|  | Putative Monogalactosyl diacylglycerol (14:0/16:1) | Galactoglycerolipids |
|  | Salmeterol | Ethanolamines |
|  | Serrawettin W1 / Serratamolide A | Depsipeptides |
|  | Soraphen A | Macrolides |
| *Ps. Acuminate*  Mature leaves | 5-epi-ilimaquinone | benzoquinones |
|  | Hyperoside | Flavonols |
|  | Benzocaine | Benzoates |
|  | Putative Monogalactosyl diacylglycerol (14:0/16:1) | Galactoglycerolipids |
| *Ps. Acuminate*  Young leaves treated with jasmonic acid | 5-epi-ilimaquinone | benzoquinones |
|  | Hyperoside | Flavonols |
|  | Benzocaine | Benzoates |
|  | Imidocarb dipropionate | Carbanilides |
|  | Karakoline | Alkaloids |
|  | Putative Monogalactosyl diacylglycerol (14:0/16:1) | Galactoglycerolipids |
|  | Putative Monogalactosyl Diacylglycerol (MGDG); 16:0/18:1 | Galactoglycerolipids |
| *Ps. Acuminate*  Mature leaves treated with jasmonic acid | 5-epi-ilimaquinone | benzoquinones |
|  | Hyperoside | Flavonols |
|  | Salmeterol | Ethanolamines |
|  | Benzocaine | Benzoates |
|  | Karakoline | Alkaloids |
|  | Putative Digalactosyl diacylglycerol (14:0/16:0) | Glycosyldiacylglycerols |
|  | Putative Monogalactosyl diacylglycerol (14:0/16:1) | Galactoglycerolipids |
|  | Sphingomyelin (18:1/14:0) | Glycolipids |
