# Supplementary material for: A comparison of inducible, ontogenetic, and interspecific sources of variation in the foliar metabolome in tropical trees
Source: PeerJ. 2019 Sep 20;7:e7536. doi: 10.7717/peerj.7536 (PMC6756142; doi:10.7717/peerj.7536)
Supplement: Table S2 — Volatile compounds were detected using GC-MS and identified by comparing Kovats retention indices to the literature. [file peerj-07-7536-s002.docx]

Table S2. Volatile compounds found in young and mature leaves of each species. Volatile compounds were detected using GC-MS and identified by comparing Kovats retention indices to the literature.

| Species | Young | Mature |
| --- | --- | --- |
| *I. cocleensis* | 1-ethyl-3-methyl-Benzene  1-Hexanol 1-Octanol 1-Octen-3-ol  1-Penten-3-one 1,3,5-trimethyl-benzene  2-ethyl-1-hexanol 2-Furanmethanol  2-methylacetophenone 2-nonenal 2,2-dimethyl-butane 3-methoxybenzyl alcohol  4-carene 4-hydroxy-4-methyl-2-pentanone  4-terpineol 5-methyl-2(5H)-furanone  6,10-dimethyl-5,9-undecadien-2-one Acetic acid Acetic acid, methyl ester  α-caryophyllene  α-cubebene α-methyl-benzeneacetaldehyde α-phellandrene α-terpineol  Benzeneacetaldehyde β-Cyclocitral β-Ionone Butyrolactone  Caryophyllene Corylone Decanal Formic acid, octyl ester  Hexadecanoic acid Hexanal Jasmone Linalol  Menthacamphor Methyl Salicylate methyl-Cyclohexane Nonanal  Octadecanoic acid p-Cymene propyl-Benzene Sabinene  Tetradecanal Tridecanal Undecanal | 1-ethyl-3-methyl-Benzene  1-Hexanol 1-Octen-3-ol 1-Penten-3-one  1,2-dimethoxy-Benzene 1,3,5-trimethyl-Benzene  2-ethyl-1-Hexanol 2-Furanmethanol  2-methyl-1,3-Butadiene  2-Methylacetophenone 3-Methoxybenzyl alcohol  3,7-dimethyl-2,6-Octadienal  4-Carene 4-terpineol 5-methyl-2(3H)-Furanone  5-methyl-2(5H)-Furanone  6-Methyl-5-heptene-2-one  6,10-dimethyl-5,9-Undecadien-2-one Acetic acid Acetic acid, methyl ester  Acetone alpha-Caryophyllene alpha-Cubebene alpha-Phellandrene  Benzeneacetaldehyde beta-Cyclocitral beta-Ionone Butyrolactone  Cyclohexanol Decanal Hexadecanoic acid Hexanal  Isomenthol methoxy-Benzene Methyl Salicylate methyl-Cyclohexane  n-Hexadecanoic acid Nonanal o-Methylacetophenone Pentanal  propyl-Benzene Sabinene Tetradecanal Tridecanal  Undecanal |
| *Pi. cordulatum* | 1-Acetylcyclohexene 1-ethyl-3-methyl-Benzene  1-Penten-3-one 1-Undecanol 1,3,5-trimethyl-Benzene  2-Heptanone 2-Nonanone 2-Nonenal  2-Undecanone 3-Carene  4-Carene  4-terpineol  6-Methyl-α-ionone Acetic acid Acetone  allo-Aromadendrene α-Caryophyllene α-Cedrene  α-Cubebene  α-Guaiene  α-Gurjunene  α-Longicyclene  α-Longifolene  α-Longipinene  α-Phellandrene  α-Phenylpropionaldehyde α-Pinene  α-Terpinene  α-Terpinenol  α-Terpineol acetate  α-Tricyclene Aristolene Aromadendrene Azulene Benzeneacetaldehyde  β-Caryophyllene epoxide  β-Cedrene  β-Cyclocitral  β-Gurjunene  β-Linalool  β-Myrcene  β-Pinene  β-Selinene Butanal, 3-methyl- Camphene  Carotol Caryophyllene Copaene Cryptone Cyclene  Decanal Decane  δ-Cadinene  δ-Caryophyllene δ-Guaiene  Diacetone alcohol Dihydrocarveol Ethylbenzene γ-Gurjunene  γ-Terpinen  γ-Terpinene Globulol Guaiol Heptadecane Heptanal  Hexanal Isolimonene Ledene Limonene Linalol  Linalyl acetate Longifolene methyl-Cyclohexane Nerolidol Nonanal  o-Xylene  p-Cymene p-Xylene Propanal, 2-methyl- propyl-Benzene  Sabinene Sativene Tridecane  Valencene  Virdiflorene | 1-ethyl-3-methyl-Benzene 1-Hexanol 1,3,5-trimethyl-Benzene 2-Acetyltoluene  2-Nonanone  2-Nonenal 3-Methoxybenzyl alcohol 4-Carene 6-Methyl-5-heptene-2-one  Acetic acid  allo-Aromadendrene α-Caryophyllene α-Cubebene  α-Guaiene  α-Gurjunene  α-Longifolene  α-Longipinene  α-Phellandrene  α-Pinene  α-Terpinenol  Aristolene Aromadendrene Azulene Benzeneacetaldehyde β-Caryophyllene epoxide  β-Cedrene  β-Cyclocitral  β-Gurjunene  β-Ionone  β-Linalool  β-Pinene  β-Selinene Camphene Caryophyllene Caryophyllene oxide  Copaene Cumene Cyclohexanol δ-Cadinene  δ-Guaiene  γ-Gurjunene  γ-Terpinen Globulol Guaiol Hexanal  Isoestragole Limonene Linalol Linalyl propionate Longifolene  Methyl Salicylate methyl-Cyclohexane Nerolidol  o-Xylene  p-Cymene  p-Xylene Phenylethyl Alcohol propyl-Benzene Propylene Glycol Sabinene  Sativene Tridecane Valencene |
| *Pr. panamense* | 1-ethyl-3-methyl-Benzene  1-Hexanol 1,3,5-trimethyl-Benzene  3-Carene  3-Isopropylbenzaldehyde 4-Carene 4-terpineol allo-Aromadendrene  α-Caryophyllene α-Cubebene α-Guaiene α-Gurjunene  α-Longicyclene α-Longipinene α-Phellandrene α-Pinene  α-Terpinene α-terpinenol α-Terpinenol α-Tricyclene  Aristolene Aromadendrene Benzeneacetaldehyde β-Cyclocitral  β-Gurjunene β-Ionone β-Linalool β-Myrcene  β-Pinene β-Selinene β-Thujone/Isothujone/(E)-Thujone Carvacrol  Caryophyllene Caryophyllene oxide Copaene Cryptone  δ-Cadinene δ-Guaiene Dihydrocarveol γ-Gurjunene  γ-Terpinen Hexanal Limonene m-xylene  Methyl Salicylate o-Xylene p-Cymene Paracymene  Sabinene Sativene Thymol Valencene | 1-ethyl-3-methyl-Benzene  1-Hexanol 1-Octen-3-ol 1,3-dimethyl-Benzene/m-xylene 1,3,5-trimethyl-Benzene  3-Carene 4-Carene 4-terpineol allo-Aromadendrene α-Caryophyllene  α-Cubebene α-Guaiene α-Gurjunene α-Longicyclene α-Longifolene  α-Longipinene α-Phellandrene α-Pinene α-Terpinene α-Terpinenol  α-Terpinolene α-Tricyclene anisole Aristolene Aromadendrene  Azulene Benzaldehyde Benzeneacetaldehyde Benzyl Alcohol β-Cyclocitral  β-Gurjunene β-Ionone β-Myrcene β-Pinene β-Santalol  β-Selinene Camphene Carvacrol Caryophyllene Copaene  Cryptone δ-Cadinene δ-Guaiene Dihydrocarveol γ-Gurjunene  γ-Hexalactone γ-Terpinen Globulol Heptanal Hexanal  Isolimonene Limonene Methyl Salicylate methyl-Cyclohexane Nonanal  o-Xylene p-Cymene Paracymene Sabinene Sativene  Thujopsene Valencene |
| *Ps. acuminata* | 1-ethyl-3-methyl-Benzene  1-Hexanol  1-methylethyl-Benzene 1-Octen-3-ol  1-t-Butyl-3-ethylbenzene  1,3,5-trimethyl-Benzene  2-ethyl-1-Hexanol  2-Nonenal  3,7-dimethyl-1-Octanol 4-Carene  4-terpineol  allo-Aromadendrene  α-Caryophyllene  α-Cedrene  α-Cubebene  α-Gurjunene  α-Phellandrene  α-Pinene  α-Terpinene  α-Terpinolene Anethole Aristolene Aromadendrene Benzaldehyde  Benzeneacetaldehyde β-Cyclocitral  β-Gurjunene  β-Ionone  β-Myrcene Butylated Hydroxytoluene  Camphor Caryophyllene Cedrol Copaene Decanal  δ-Cadinene  δ-Guaiene Ethylbenzene  γ-Gurjunene  γ-Terpinene Hexanal Linalol  Linalyl acetate Menthacamphor Methyl Salicylate methyl-Cyclohexane Nonanal  o-Xylene  p-Cymene  p-Xylene Pantolactone propyl-Benzene Sabinene trans-β-lonone  Valencene | 1-ethyl-3-methyl-Benzene  1-Nonanol 1-Octen-3-ol 1-Penten-3-one  1,3,5-trimethyl-Benzene  2-ethyl-1-Hexanol 2-methoxy-6-methyl-Pyrazine  2-Methoxy-6-methylpyrazine  2-Methylacetophenone 2,2-dimethyl-Butane 3-Methoxybenzyl alcohol  4-hydroxy-4-methyl-2-Pentanone  6-Methyl-5-heptene-2-one  Acetic acid α-methyl-Benzeneacetaldehyde α-Terpineol  Benzaldehyde Benzeneacetaldehyde β-Cyclocitral β-Ionone  Butyl isobutyrate Camphor Caryophyllene Decanal  δ-Guaiene Ethylbenzene Heptanal Hexanal  Linalol Methyl Salicylate methyl-Cyclohexane Nonanal  o-Xylene Pantolactone propyl-Benzene Sabinene |
