# Supplementary figures and images for: A comparison of inducible, ontogenetic, and interspecific sources of variation in the foliar metabolome in tropical trees
Source: PeerJ. 2019 Sep 20;7:e7536. doi: 10.7717/peerj.7536 (PMC6756142; doi:10.7717/peerj.7536)

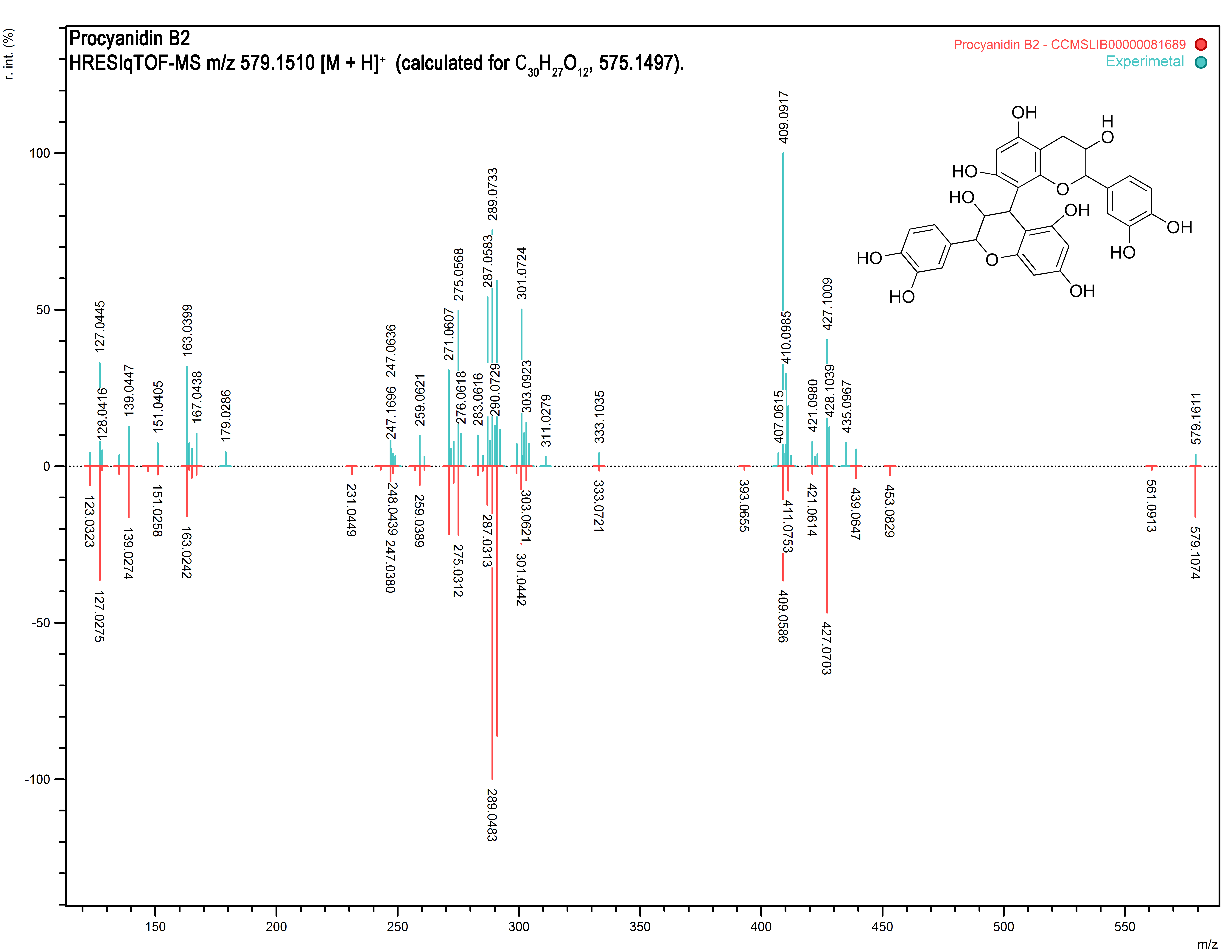

Supplement: Supplemental Information 1 — Annotated Spectra for compounds that matched records in Global Natural Products Social (GNPS) Molecular Networking mass spectra libraries with cosine score above 0.8 (http://gnps.ucsd.edu ID=6d2ad31f795d4975b3d22675b4d24cac). Each spectrum collected using a high resolution q-TOF mass spectrometer with an electrospray ionization source (HRESIqTOF-MS) was compared with GNPS database spectra using mirror view with mMass (version 5.5.0), followed by evaluation of the major collision induced dissociation (CID) as described by Demarque et al. (DOI: 10.1039/c5np00073d), and finally molecular formula was calculated using precursor ion mass with Bruker Compass Data Analysis 4.1. [file peerj-07-7536-s003.png]

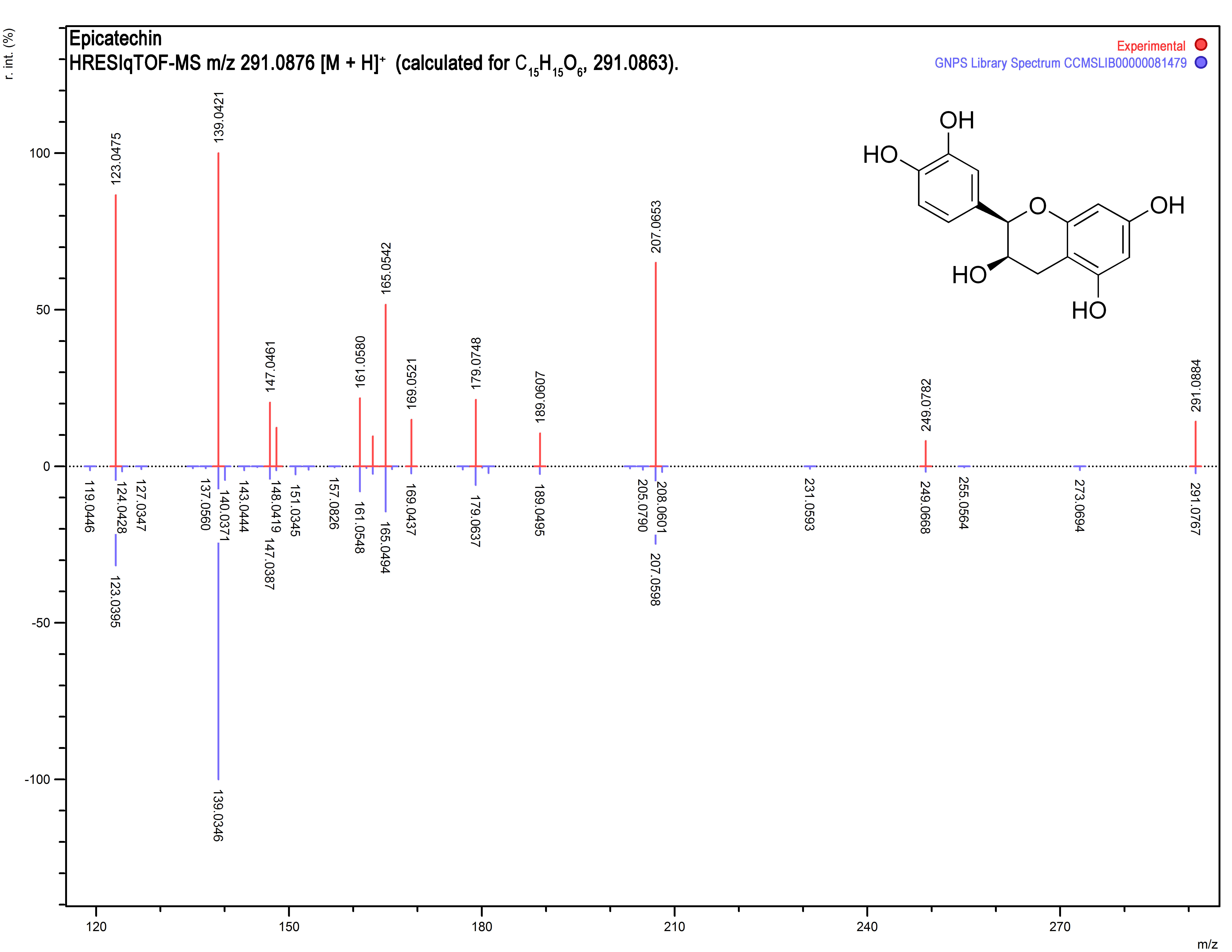

Supplement: Supplemental Information 2 — Annotated Spectra for compounds that matched records in Global Natural Products Social (GNPS) Molecular Networking mass spectra libraries with cosine score above 0.8 (http://gnps.ucsd.edu ID=6d2ad31f795d4975b3d22675b4d24cac). Each spectrum collected using a high resolution q-TOF mass spectrometer with an electrospray ionization source (HRESIqTOF-MS) was compared with GNPS database spectra using mirror view with mMass (version 5.5.0), followed by evaluation of the major collision induced dissociation (CID) as described by Demarque et al. (DOI: 10.1039/c5np00073d), and finally molecular formula was calculated using precursor ion mass with Bruker Compass Data Analysis 4.1. [file peerj-07-7536-s004.png]

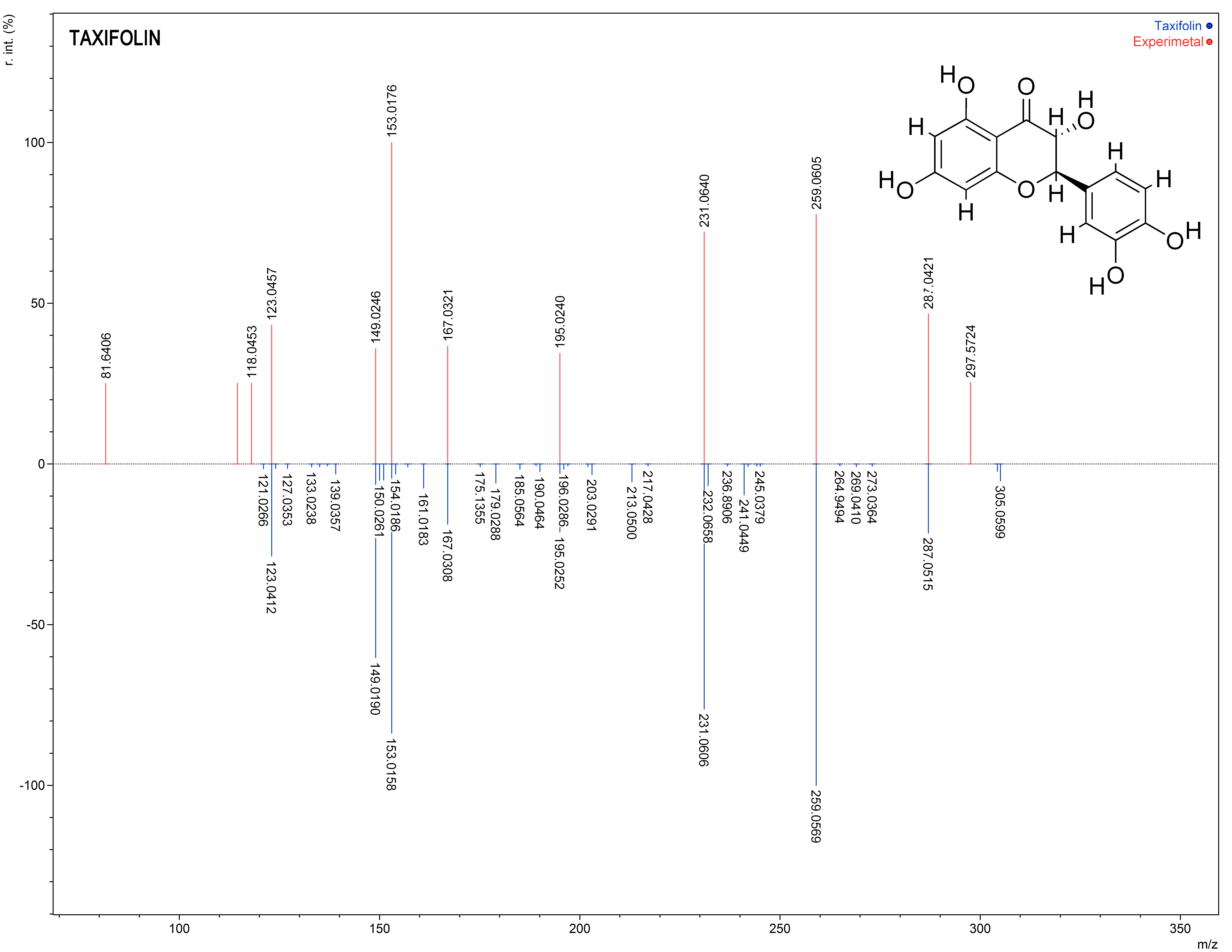

Supplement: Supplemental Information 3 — Annotated Spectra for compounds that matched records in Global Natural Products Social (GNPS) Molecular Networking mass spectra libraries with cosine score above 0.8 (http://gnps.ucsd.edu ID=6d2ad31f795d4975b3d22675b4d24cac). Each spectrum collected using a high resolution q-TOF mass spectrometer with an electrospray ionization source (HRESIqTOF-MS) was compared with GNPS database spectra using mirror view with mMass (version 5.5.0), followed by evaluation of the major collision induced dissociation (CID) as described by Demarque et al. (DOI: 10.1039/c5np00073d), and finally molecular formula was calculated using precursor ion mass with Bruker Compass Data Analysis 4.1. [file peerj-07-7536-s005.png]
